# Supplementary figures and images for: A smart tele-cytology point-of-care platform for oral cancer screening
Source: PLoS One. 2019 Nov 15;14(11):e0224885. doi: 10.1371/journal.pone.0224885 (PMC6857853; doi:10.1371/journal.pone.0224885)

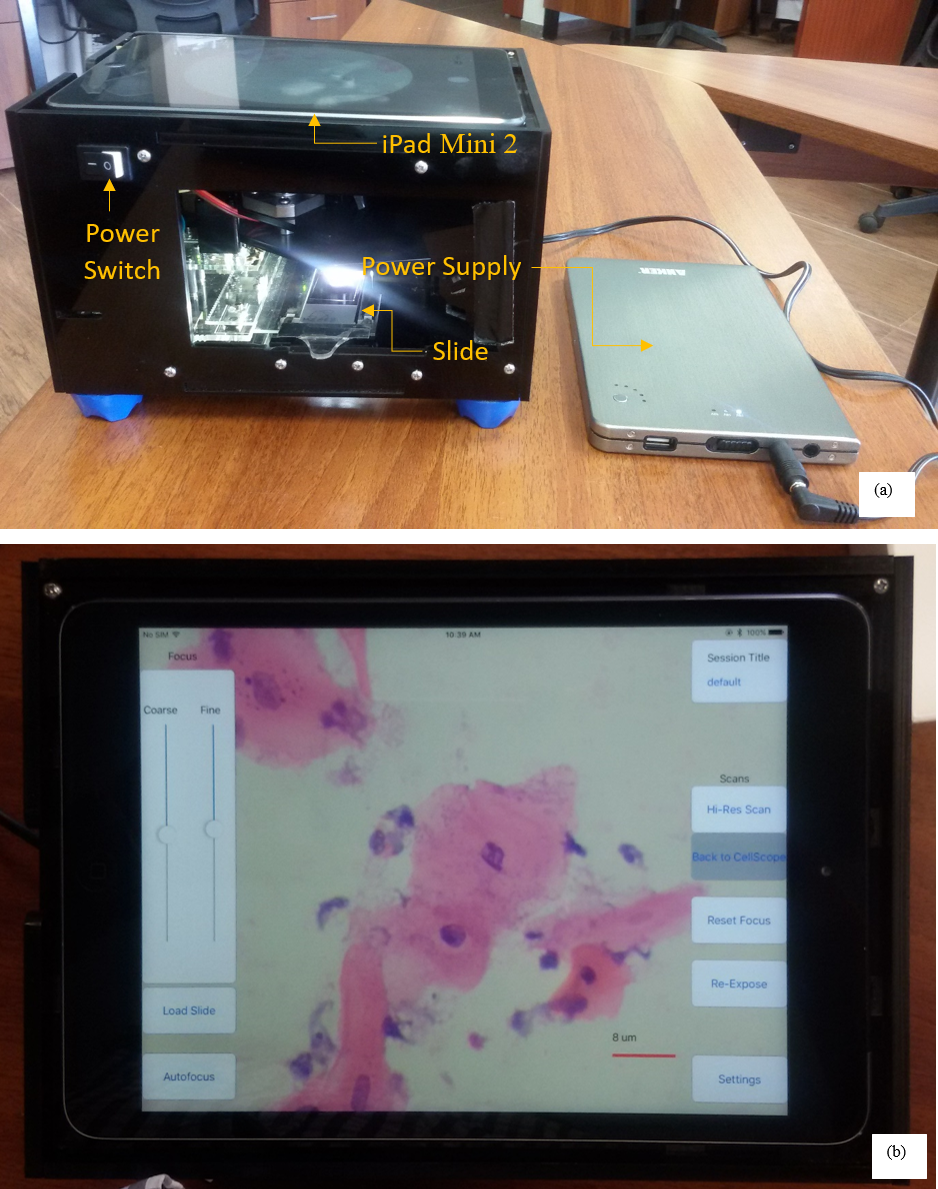

Supplement: S1 Fig — Front View of the device (A) showing the scanning platform and top view showing the iPad mini 2 (B) as the user interface. (TIF) [file pone.0224885.s001.tif]

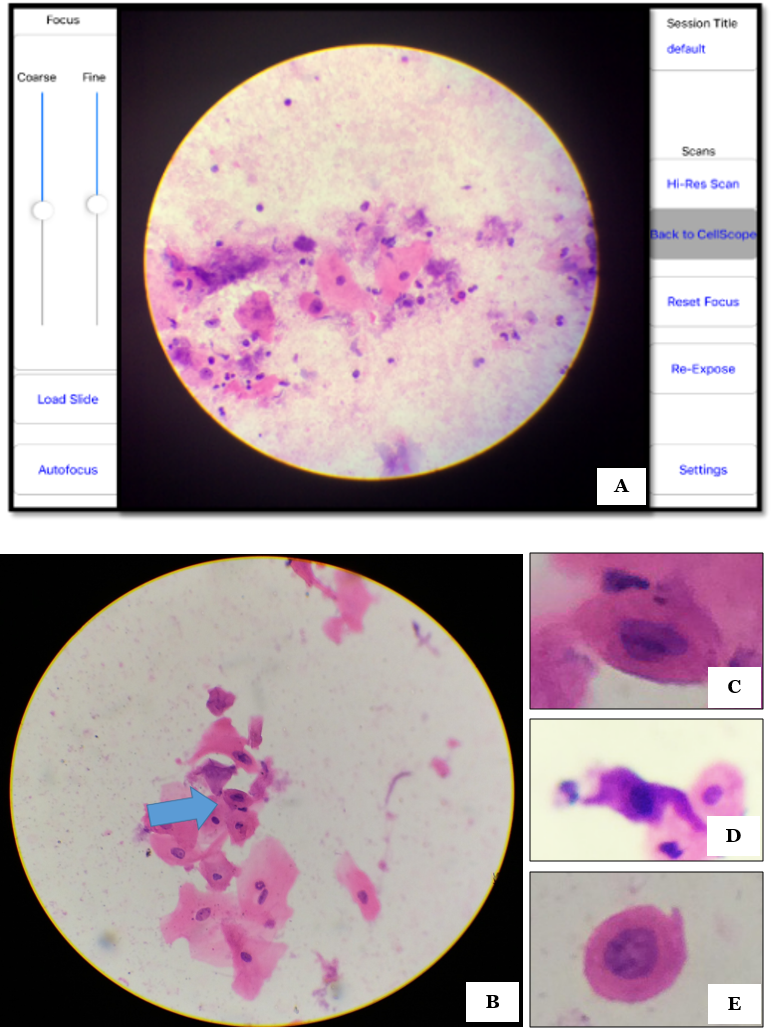

Supplement: S2 Fig — An image, (A) captured by Cellscope (200X magnification) with resolution of 2592 x 1936 (72dpi) and cell with irregular nuclear membrane, (B, blue arrow). The images were zoomed in to 200% representing cell with irregular nuclear membrane, (C), abnormal cell shape, (D) and increased nuclear to cytoplasmic ratio, (E). (TIF) [file pone.0224885.s002.tif]

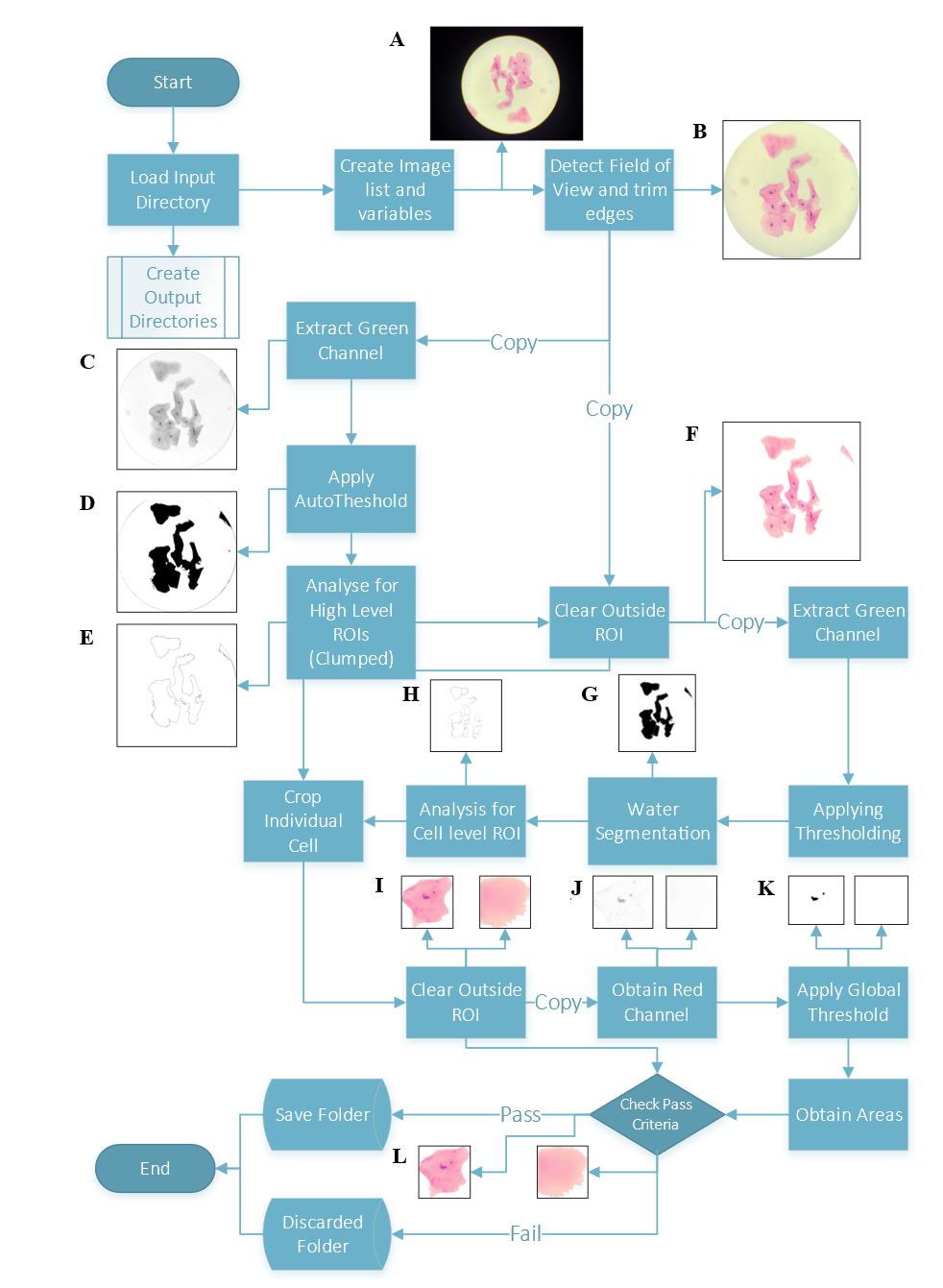

Supplement: S3 Fig — An Input image, (A) was analysed to estimate the Field of View (FOV) and was trimmed to contain only the same, (B), once detected, the green channel from RGB image, (C) was considered to detect areas of cellular mass owing to its better contrast of cell mass. These images were thresholded, (D) to get cellular area. The binarized image is then analysed for extracting high level Region of Interests (ROI), (E) that is used to clear the background, (F). Images were then water segmented, (G) and again analysed for cellular-level ROIs, (H). Then each ROI, (I) is then extracted as a single image. The red channel, (J), is then obtained and thresholded, (I) to detect nucleus. The images are then checked with pass criteria and then saved, (L) into a folder. (TIF) [file pone.0224885.s003.tif]

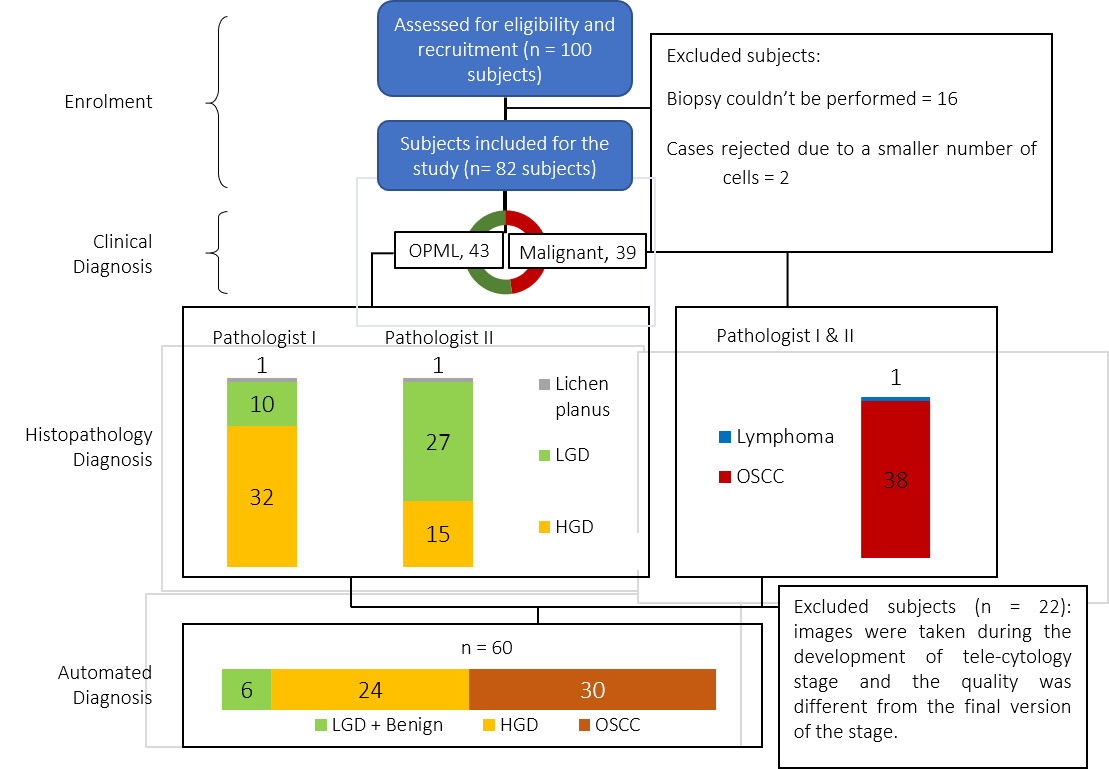

Supplement: S4 Fig — Distribution of subjects according to clinical and histopathological diagnosis. Eighty subjects were included in the analysis, of which, 43 were OPML and 39 were malignant. For automated diagnosis, 22 subjects were excluded since their images were taken during the developmental stages of the tele-cytology platform and the images were very different from the final set of images. Thus (n = 60) subjects were considered for development and validation of ANN based diagnosis. OPML = Oral Potentially Malignant Lesion, HGD = High Grade Dysplasia, LGD = Low Grade Dysplasia, OSCC = Oral Squamous Cell Carcinoma. (TIF) [file pone.0224885.s004.tif]

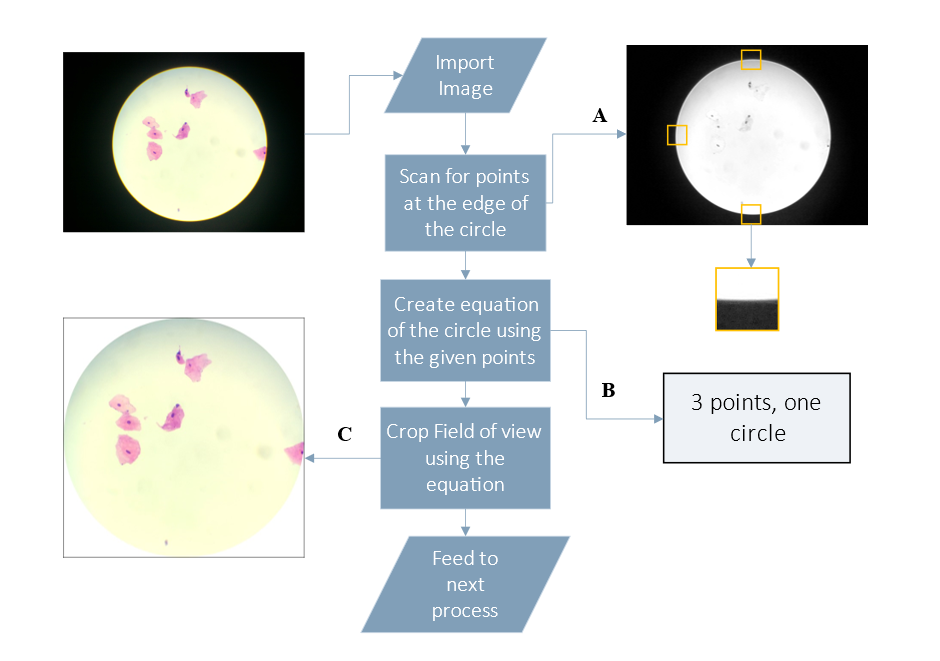

Supplement: S5 Fig — The algorithm finds out 3 pixels at the edge of the FOV in the input image, (A), Assuming the FOV is circular, the equation of circle (x−p)2+(x−p)2 = r2 representing the circular edge is solved to obtain the boundary of ROI, (B), a circular ROI is then extracted, (C). (TIF) [file pone.0224885.s005.tif]

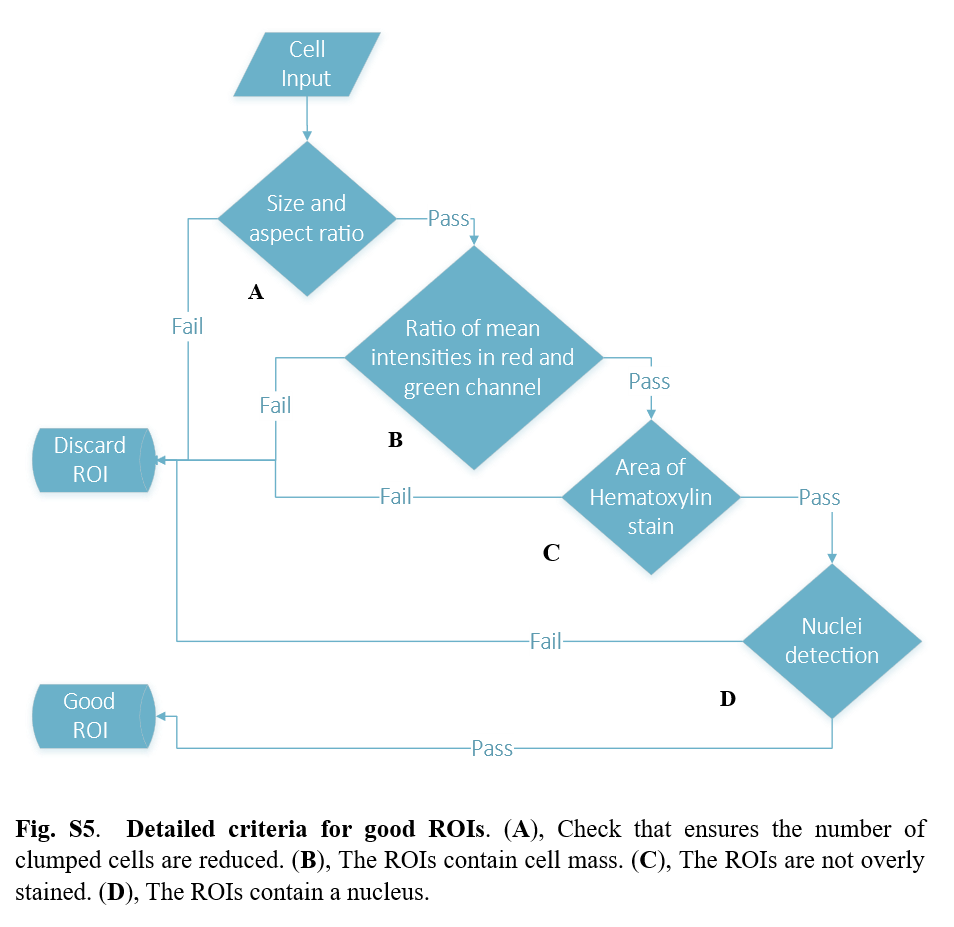

Supplement: S6 Fig — The first test (A) reduces the number of clumped cells based on size and aspect ratio. From the passed images, images with shadow artefacts are removed based on the ratio of mean channel intensities of red and green channels, (B), which are then again filtered based on Hematoxylin stained area, (C). Finally, the ROIs are analysed to find the presence of a nucleus, (D). (TIF) [file pone.0224885.s006.tif]

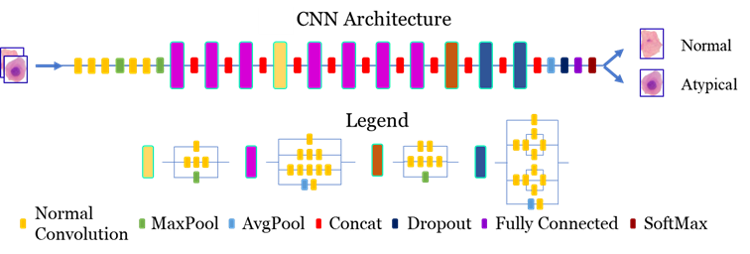

Supplement: S7 Fig — The deep convolutional neural network used here to delineate between normal cells and atypical cells. (TIF) [file pone.0224885.s007.tif]
