# Supplementary material for: A smart tele-cytology point-of-care platform for oral cancer screening
Source: PLoS One. 2019 Nov 15;14(11):e0224885. doi: 10.1371/journal.pone.0224885 (PMC6857853; doi:10.1371/journal.pone.0224885)
Supplement: S1 Table — (DOCX) [file pone.0224885.s008.docx]

| Sex | | | Age | | | Risk Habits | | |
| --- | --- | --- | --- | --- | --- | --- | --- | --- |
| Criteria | n | % | Criteria | n | % | Criteria | n | % |
| Male | 64 | 78% | >70 | 2 | 2 | None | 13 | 16 |
| Female | 18 | 22% | 61-70 | 16 | 20 | Tobacco | 61 | 74.3 |
| Total | 82 | 100% | 51-60 | 13 | 16 | Drinking | 1 | 1.2 |
|  |  |  | 41-50 | 18 | 22 | Both | 7 | 8.5 |
|  |  |  | 31-40 | 21 | 26 |  | 82 | 100 |
|  |  |  | 21-30 | 12 | 14 |  | | |
|  |  |  | Total | 82 | 100 |  |  |  |

**S1 Table: Showing demographics of subjects** Sex, age and risk habits of the subjects
