# Supplementary material for: A smart tele-cytology point-of-care platform for oral cancer screening
Source: PLoS One. 2019 Nov 15;14(11):e0224885. doi: 10.1371/journal.pone.0224885 (PMC6857853; doi:10.1371/journal.pone.0224885)
Supplement: S2 Table — Comparison of performance of various machine learning models used to delineate Oral Squamous Cell Carcinoma (OSCC) from High Grade Dysplasia (HGD) and Low-Grade Dysplasia (LGD). (DOCX) [file pone.0224885.s009.docx]

| S1 Table. Comparison of performance of various machine learning models used to delineate Oral Squamous Cell Carcinoma (OSCC) from High Grade Dysplasia (HGD) and Low-Grade Dysplasia (LGD), n=30 | | | | | |
| --- | --- | --- | --- | --- | --- |
|  | **SVM** | **Random forest** | **Logistic regression** | **LDA** | **KNN** |
| True positive | 14 | 14 | 12 | 12 | 11 |
| True negative | 13 | 12 | 13 | 13 | 12 |
| False positive | 2 | 3 | 2 | 2 | 3 |
| False negative | 1 | 1 | 3 | 3 | 4 |
| Sensitivity | 93% | 93% | 80% | 80% | 73% |
| Specificity | 88% | 80% | 87% | 87% | 80% |
| Accuracy | 90% | 87% | 83% | 83% | 77% |
